# Integrative BNN-LHS Surrogate Modeling and Thermo-Mechanical-EM Analysis for Enhanced Characterization of High-Frequency Low-Pass Filters in COMSOL †

**DOI:** 10.3390/mi15050647

**Published:** 2024-05-13

**Authors:** Jorge Davalos-Guzman, Jose L. Chavez-Hurtado, Zabdiel Brito-Brito

**Affiliations:** 1 Intel Corporation, Folsom, CA 95630, USA; jorge.davalos.guzman@intel.com; 2 Department of Electronics, Systems, and Informatics, ITESO (*Instituto Tecnológico y de Estudios Superiores de Occidente*), The Jesuit University of Guadalajara, Tlaquepaque 45604, Jalisco, Mexico; 3 Centre Tecnològic de Telecomunicacions de Catalunya (CTTC/CERCA), Castelldefels, 08860 Barcelona, Spain; zbrito@cttc.es

**Keywords:** Bayesian Neural Networks (BNNs), electromagnetic (EM) analysis optimization, Latin Hypercube Sampling (LHS) techniques, advanced low-pass filter simulation, COMSOL multiphysics predictive modeling, thermo-electromagnetic behavior analysis, high-dimensional data analysis in electronics, variability sensitivity in electronic filters, machine learning applications in EM design, data-driven design and simulation enhancement

## Abstract

This paper pioneers a novel approach in electromagnetic (EM) system analysis by synergistically combining Bayesian Neural Networks (BNNs) informed by Latin Hypercube Sampling (LHS) with advanced thermal–mechanical surrogate modeling within COMSOL simulations for high-frequency low-pass filter modeling. Our methodology transcends traditional EM characterization by integrating physical dimension variability, thermal effects, mechanical deformation, and real-world operational conditions, thereby achieving a significant leap in predictive modeling fidelity. Through rigorous evaluation using Mean Squared Error (MSE), Maximum Learning Error (MLE), and Maximum Test Error (MTE) metrics, as well as comprehensive validation on unseen data, the model’s robustness and generalization capability is demonstrated. This research challenges conventional methods, offering a nuanced understanding of multiphysical phenomena to enhance reliability and resilience in electronic component design and optimization. The integration of thermal variables alongside dimensional parameters marks a novel paradigm in filter performance analysis, significantly improving simulation accuracy. Our findings not only contribute to the body of knowledge in EM diagnostics and complex-environment analysis but also pave the way for future investigations into the fusion of machine learning with computational physics, promising transformative impacts across various applications, from telecommunications to medical devices.

## 1. Introduction

In the face of escalating complexity in electromagnetic (EM) system design and the pressing demand for advanced simulation tools, our study embarks on a pioneering journey at the nexus of machine learning and computational physics to redefine EM characterization. Central to our investigation is the innovative integration of Bayesian Neural Networks (BNNs) with Latin Hypercube Sampling (LHS) within COMSOL simulations, targeting the analysis of coarse and fine variable-dimension low-pass filters. This dual-pronged approach not only forecasts EM behavior alterations due to physical dimension variability, but also propels system modeling optimization through sophisticated neural networks. The amalgamation of BNNs and LHS heralds a groundbreaking methodology in EM analysis, promising a pathway to achieve unparalleled accuracy in simulations while optimizing computational efficiency [1,2,3,4].

Our research transcends traditional computational physics through the lens of machine learning’s predictive prowess, facilitating a deeper comprehension of EM behavior amidst varying physical dimensions and streamlining the design and optimization of EM systems. By marrying the strengths of both domains, we confront the challenges posed by the intricate complexity of EM systems, delivering a robust framework for the precise and efficient analysis of low-pass filters [5,6,7]. The hallmark of our methodology lies in its ability to maintain the granularity of conventional simulations while significantly diminishing computational demands, a critical factor for expediting the release of design iterations and prototype development [8,9].

At the heart of our analysis is the S21 parameter, a crucial metric of power transmission in filters, whose variability under diverse geometric alterations is meticulously evaluated to underscore our model’s sophisticated understanding [10,11]. Through an exhaustive parameter sweep, our research unveils the subtle impacts of minute dimensional adjustments, offering a rich, comprehensive analysis that is both thorough and computationally savvy. The ramifications of this endeavor are vast, enriching electronic design optimization and heralding a new epoch of data-driven diagnostics within complex EM landscapes [12,13,14].

Looking ahead, this paper ventures into advanced data-driven methodologies like Bayesian optimization [15], further honing the acuity of our BNN model. Such strategies signal a future where electronic design is not merely data-informed but markedly more efficient, marking a significant stride in melding machine learning with computational physics to navigate the evolving challenges in EM system design and optimization.

This investigation also introduces an avant-garde thermal optimization framework that leverages BNN-enhanced with LHS and temperature variables, setting a new paradigm for low-pass filter modeling. This integration of temperature as a pivotal variable alongside dimensional parameters, operational frequency, and S-parameter values ushers in a multiphysical approach in filter performance analysis, embodying a paradigm shift towards simulating real-world operational conditions with unmatched precision [16,17,18]. Our findings, buttressed by rigorous validation of the BNN model on unseen data, promise transformative impacts in EM simulation and design, promoting a data-driven design and simulation enhancement to significantly uplift the accuracy and efficiency of predictive modeling [19,20,21,22].

## 2. Evolutionary Design Optimization of Low-Pass Filters in COMSOL: Bridging Baseline Electromagnetic Analysis with Enhanced Multiphysics Simulations

Our investigation represents a methodical advancement in the domain of electromagnetic (EM) system design, employing a phased approach within the COMSOL multiphysics environment to model high-frequency low-pass filters. The journey begins with a baseline design, focusing on electromagnetic phenomena through a coarse mesh architecture, and progresses to an advanced multiphysics design, which incorporates a fine mesh to simulate additional thermal and mechanical dynamics. This evolutionary approach not only delineates the transition from a singular focus on EM analysis to a comprehensive multiphysics simulation but also sets a precedent for achieving unparalleled predictive modeling accuracy and computational efficiency.

### 2.1. Baseline Design: Coarse Mesh Optimization for Electromagnetic Fidelity

#### 2.1.1. Mesh Design and Justification

**Figure 1:**
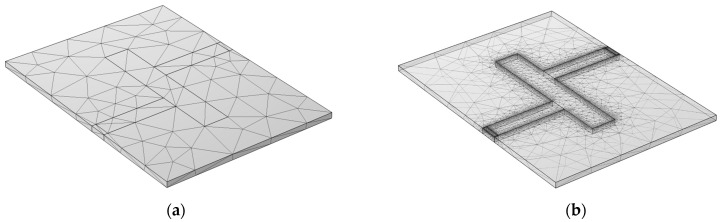
Finite element mesh of a low-pass microstrip filter for EM analysis as implemented in COMSOL: (**a**) mesh designed to optimize the resolution of electromagnetic phenomena; (**b**) mesh architecture refined to incorporate thermo-mechanical EM analysis.

The baseline design phase is foundational, emphasizing the critical role of mesh architecture in simulation fidelity. The coarse mesh is illustrated in Figure 1a.

The coarse mesh was intricately engineered to enhance the resolution of electromagnetic phenomena. Special attention was given to interfaces anticipated to exhibit significant field gradients, particularly near microstrip lines and port regions, where boundary conditions induce complex field interactions. This strategic densification of mesh elements aimed to precisely characterize the filter’s response, ensuring robust model predictions across various mesh densities as shown in Table 1.

This strategic densification, especially near microstrip lines and port regions, aimed to precisely characterize the filter’s response to complex field interactions induced by boundary conditions.

#### 2.1.2. Simulation Parameters and Design Space Exploration

**Figure 2:**
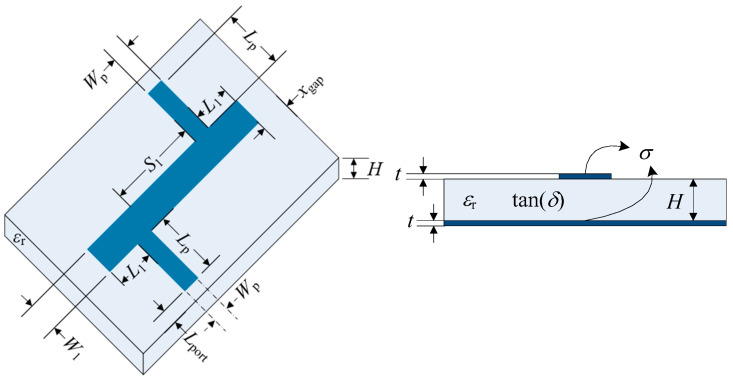
Low-pass microstrip filter geometry taken from [23], illustrating the physical dimensions of the circuit implemented in COMSOL.

Our simulation parameters were selected with dual objectives: to capture the intricate behavior of the low-pass filter within its operational bandwidth of 100 MHz to 10 GHz and to ensure the practicality of simulation runs. Mesh element sizes were meticulously tailored to the geometric complexities of the filter design. We used a duroid substrate for the structure, neglecting metallic and dielectric losses, (*δ*) = 0 and *σ =* 0 facilitating an optimized performance of the low-pass filter that has been widely studied in [23] and is depicted in Figure 2.

This structured approach, leveraging a Bayesian Neural Network (BNN) trained with Latin Hypercube Sampling (LHS) data, enabled a comprehensive exploration of the design space, ensuring a robust dataset that accurately reflected the diverse operational conditions of the filter.

#### 2.1.3. BNN Training Dataset

The BNN was trained using a dataset featuring five key variables τ = [$W_{1}$, $L_{1}$, $S_{1}$, F, $S_{21}$]. This comprehensive training set was derived from the integration of LHS and empirical data, providing a strategic balance of dimensional variables and operational frequencies, encapsulated in a dataset of 2000 entries, derived from 40 frequency points and 50 sampled regions using LHS, suggesting a well-distributed dataset over the parameter space and frequency range.

This methodology involved a parameter sweep designed to refine the filter’s design by adjusting its physical dimensions, namely, width ($W_{1}$), length ($L_{1}$), and spacing ($S_{1}$), to meticulously assess their influence on the filter’s electromagnetic behavior by executing a targeted sweep that varied from 0.1% to 0.3% for these dimensions and using the parameters in Table 2.

By ensuring a holistic simulation of the filter’s performance across the designated frequency range and under varying thermal conditions, we were able to precisely gauge their effects on filter performance at high frequencies. The performance metrics of BNN training for different configurations, measured in terms of MSE, MLE, and MTE, are shown in Table 3, along with the number of neurons used in the network.

**Figure 3:**
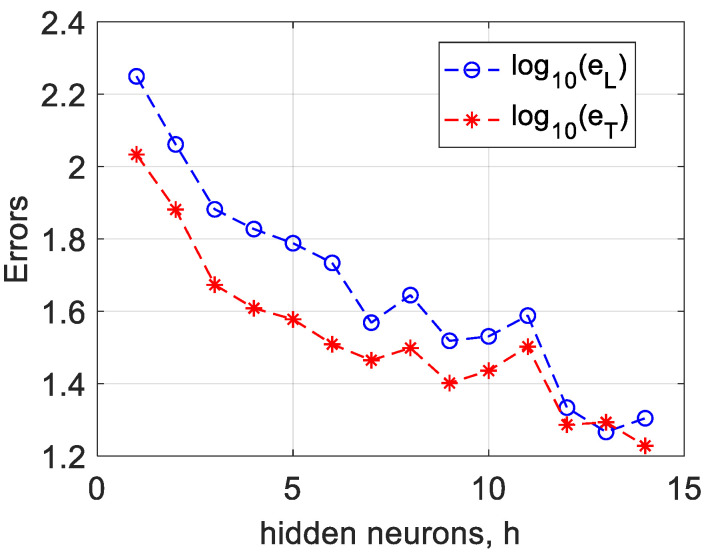
Corresponding learning and testing error dynamics from hidden neurons for 0.15 variation.

Figure 3 illustrates the logarithmic scale of learning and testing errors as a function of the number of hidden neurons in the network.

The integration of the LHS method with this parameter sweep facilitated a nuanced exploration of the design space, ensuring the generation of a robust dataset that accurately reflected the diverse operational conditions of the filter. This comprehensive approach not only enhanced the predictive accuracy of our simulations but also contributed to the ongoing refinement of low-pass filter design, embodying a synergy between computational efficiency and nuanced understanding of electromagnetic phenomena.

### 2.2. Phase II: Advanced Multiphysics Design Integrating Fine Mesh for EM, Thermal, and Mechanical Simulations

The advanced multiphysics design phase introduces a fine mesh architecture that accounts for thermal influences alongside electromagnetic analysis. This refinement, depicted in Figure 1b, aims to simulate real-world operational conditions with heightened accuracy, capturing the filter’s response to temperature fluctuations and mechanical stresses. Retaining the core structure of the initial methodology, this phase expands the focus to include thermal aspects of filter performance. The rationale for integrating temperature into the parameter sweep is articulated, emphasizing its significance in enhancing overall simulation accuracy. This methodological expansion is critical for developing filters that exhibit robust performance across diverse environmental conditions.

### 2.3. Advanced BNN Training Incorporating Temperature Dynamics

The training regimen for the BNN encompassed temperature as a critical input parameter, alongside physical dimensions and frequency. The dataset size and composition were expanded to ***x*** = [$W_{1}$, $L_{1}$, $S_{1}$, T, F, $S_{21}$]. This including temperature dynamics, which were pivotal for the BNN’s ability to generalize across different thermal conditions and design modifications.

### 2.4. Implications of Temperature in Modeling

With temperature being a variable in the BNN training, the model navigated a more complex predictive terrain. Our analysis indicated a critical intersection where neuron count and thermal factors interact, suggesting a sweet spot where temperature-induced variabilities are effectively captured without overfitting, as evidenced by the minimal MSE at the 0.1-sampled configuration in Table 4.

The findings from the 0.1-sampled BNN offer an empirical benchmark for future modeling endeavors. The lowest MSE observed suggests that with careful calibration, a BNN can account for both dimensional and thermal variabilities with high fidelity, setting the stage for a comprehensive approach to filter optimization that is both robust and sensitive to a spectrum of operational influences.

### 2.5. Neural Complexity and Error Dynamics in Multiphysical Optimization

**Figure 4:**
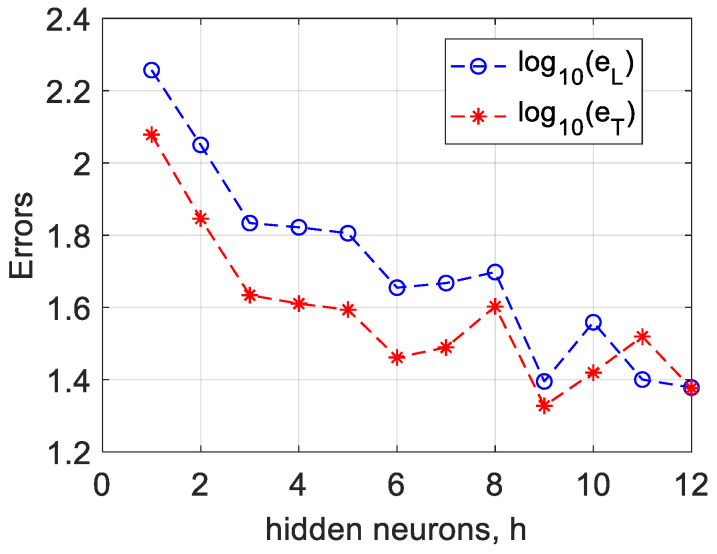
Corresponding learning and testing error dynamics from hidden neurons for 0.1 variation.

The progression of BNN sophistication within this study is represented by an advanced multiphysical framework that delineates the learning and testing error patterns as a function of hidden neuron variations, offering insights into the optimal balance between network complexity and predictive accuracy. The nuanced impact of integrating thermal dynamics into the predictive model was analyzed, identifying a sweet spot where temperature-induced variabilities were effectively captured without overfitting, illustrated by Figure 4.

## 3. Analytical Performance Evaluation

### 3.1. Phase I: Baseline Design Performance Metrics

In the initial phase of our investigation, we meticulously analyzed the performance metrics of BNN training across various configurations, focusing on MSE, MLE, and MTE, as detailed in Table 3. Our analysis revealed that the configuration with a 0.15% variation and 50 samples achieved the lowest MSE at 0.2903, suggesting an optimal balance in model training that effectively mitigated both overfitting and underfitting. This balance was crucial for enhancing predictive accuracy, particularly in configurations where the percentage variation increased, leading to a notable rise in MSE and indicating a potential decrease in model precision.

The MLE metrics further support this observation, with lower values for the 0.15% and 0.1% configurations indicating efficient learning without overfitting. Conversely, a spike in MLE at the 0.2% variation suggests challenges in model learning, potentially due to increased complexity or overfitting. Similarly, the MTE metrics underscore the model’s generalization capabilities, with the 0.15% configuration demonstrating a strong performance on unseen data. However, higher MTE values in configurations such as 0.25% and 0.3% indicate a decline in the model’s ability to generalize, likely due to the overlearning of specific patterns in the training data.

Interestingly, the number of neurons does not directly correlate with improved performance across all metrics. This observation emphasizes that the efficacy of the model is not solely dependent on network complexity but on the strategic combination of neuron count and input variation, aiming for the lowest MSE while maintaining a balance between MLE and MTE for optimal generalization.

### 3.2. Phase II: Insights from Advanced Multiphysics Design

The exploration of dimensional variations from 0.1% to 0.3% in the Advanced Multiphysics Design phase provided nuanced insights into the BNN model’s performance under diverse configurations. The general trend observed was an increase in MSE with higher variation, indicating a decrement in model accuracy, which was particularly marked by a significant spike in MSE at a 0.3% variation. This trend highlights the model’s challenges in generalizing across extensive data variations, a complexity further compounded by the introduction of temperature dynamics.

The configuration with a 0.1% variation, showcasing the lowest MSE at 0.4083, establishes a benchmark for accuracy, suggesting that minimal dimensional variation facilitates the highest levels of model accuracy. This finding is pivotal, as it sets a baseline for evaluating the impact of additional complexities, such as increased variation or temperature integration, on model performance.

The integration of temperature as a variable added a layer of complexity that significantly influenced MSE outcomes, underscoring the importance of achieving an optimal balance between dimension variation, learning efficiency, and network complexity for superior model performance. The analysis of MLE and MTE metrics further enriches our understanding, indicating that high learning efficiency does not necessarily equate to improved generalization, as reflected by elevated MSE levels.

Network complexity, as indicated by the neuron count, varied slightly across configurations, with no direct correlation to performance improvement. This observation reinforces the notion that optimal model performance is achieved through the synergy between network complexity and data variation, rather than network complexity alone.

## 4. Enhanced Model Validation and Error Dynamics for Predictive Modeling in EM System Design

**Figure 5:**
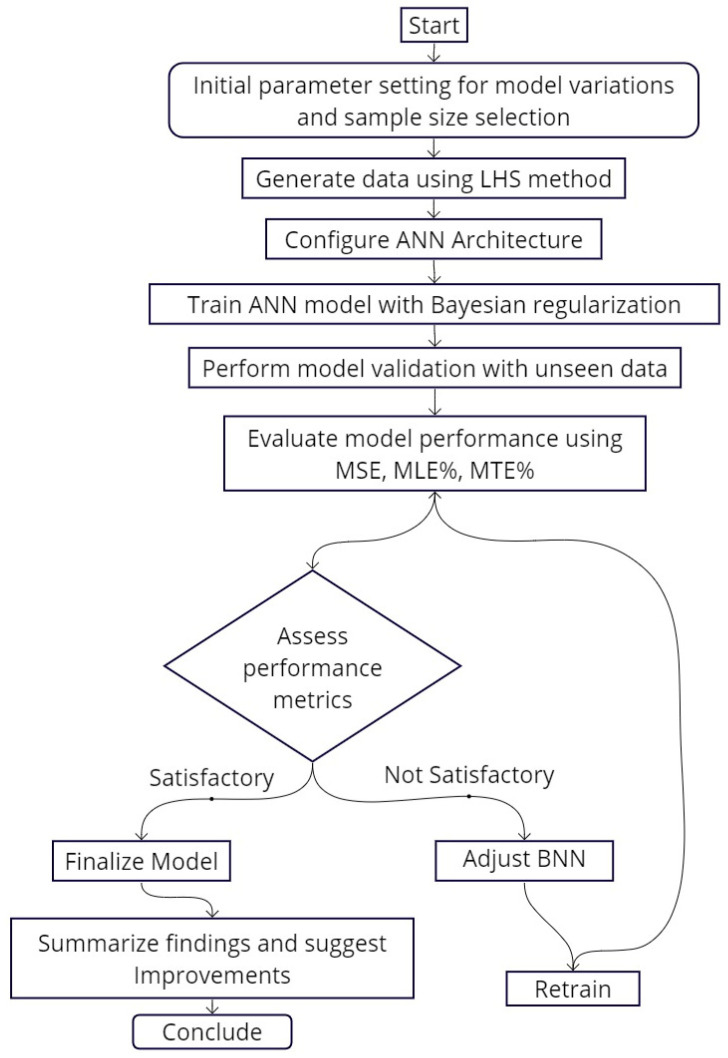
Training and validation workflow for BNN with dimensional sampling.

Our investigation into the Bayesian Neural Network (BNN) model’s validation and error analysis provides a basis for ensuring its robustness and practical applicability for electromagnetic (EM) system design and optimization. This critical evaluation process, depicted in Figure 5, is fundamental in transforming theoretical models into viable solutions that markedly enhance EM characterizations in real-world applications.

### 4.1. Coarse Baseline Design Validation

#### 4.1.1. Performance Insights and Error Distribution

**Figure 6:**
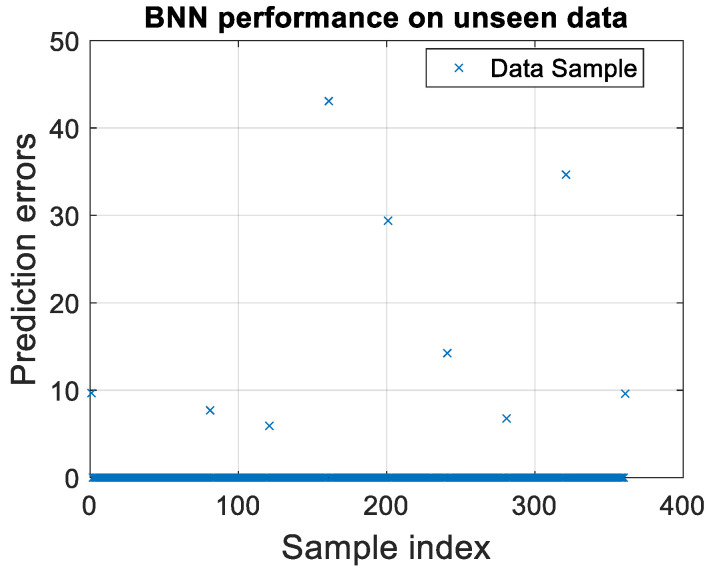
Corresponding scatter plot of predictive validation for BNN on unseen data using τ = 0.15 and 1000 sampling points.

Delving into the model’s performance through a detailed analysis of prediction errors, we identified key areas for model refinement and enhancement. This rigorous examination, vital for the model’s practical deployment, involved scrutinizing deviations from expected outcomes, as illustrated in Figure 6.

The distribution of prediction errors for unseen data samples reveals a concentration of data points towards the lower end of the prediction error scale, affirming the BNN’s efficacy in accurately forecasting most test cases. However, the presence of outliers with significantly higher prediction errors highlights instances where the model’s forecasts diverge markedly from the actual values. These outliers are invaluable for identifying the model’s limitations and areas requiring refinement.

Moreover, the consistent performance of the model across various subsets of unseen data underscores its robust generalization capability. It indicates that the BNN has successfully captured underlying patterns rather than merely memorizing the training data. The aggregation of predictions near the zero-error line for a substantial portion of the data suggests that the model’s features and architecture are aptly suited for the task. Nonetheless, regions with elevated errors signal potential overfitting issues, where the model excels with training data but falters in predicting new, unseen data. This discrepancy may stem from an overly complex model or training data that fail to represent the full spectrum of variability in unseen data.

Through this validation and error analysis, we not only affirmed the BNN model’s predictive accuracy but also illuminated pathways for its continuous improvement. These insights are crucial for refining the model, enhancing its predictive precision, and ensuring its readiness for real-world applications. The findings from this section not only demonstrate the model’s current capabilities but also lay the groundwork for future enhancements, promising a new era of efficiency and accuracy in EM analysis.

#### 4.1.2. Advanced Multiphysics Design Validation

**Figure 7:**
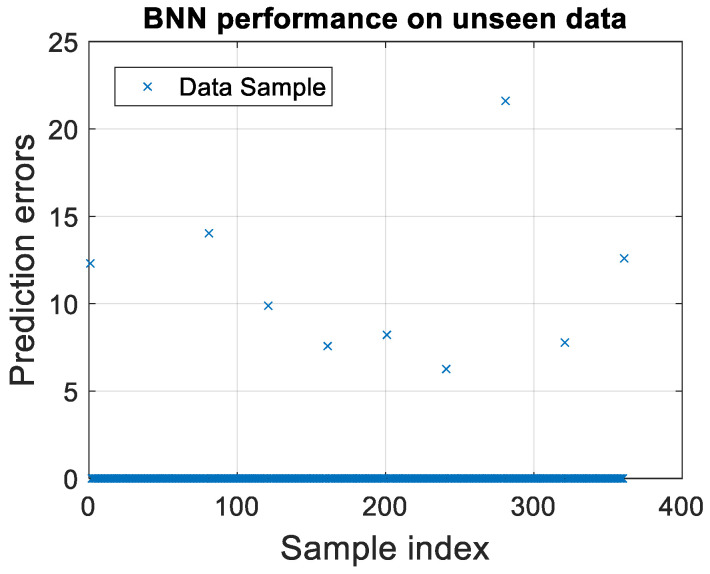
Corresponding scatter plot of predictive validation for BNN on unseen data using τ = 0.1 and 1000 sampling points.

Utilizing a robust dataset characterized by a 0.1 variation and 1000 sampling points, the scatter plot in Figure 7 provides a visual representation of the BNN’s validation process.

Each data point reflects the prediction error for individual unseen samples, offering a granular view of the model’s precision across a broad operational range.

The scatter plot indicates a predominant aggregation of lower prediction errors, suggesting that the BNN can deliver precise forecasts for most scenarios. However, outlier data points, representing higher prediction errors, warrant closer examination to fine-tune the model’s predictive algorithms and enhance its accuracy.

The validation of the BNN on unseen data is not merely a measure of model performance but also a testament to the model’s adaptability and reliability in practical applications. The insights gained from this analysis have profound implications for future EM simulations, where predictive accuracy is paramount.

## 5. Results

### 5.1. Baseline Design Comparative Analysis

**Figure 8:**
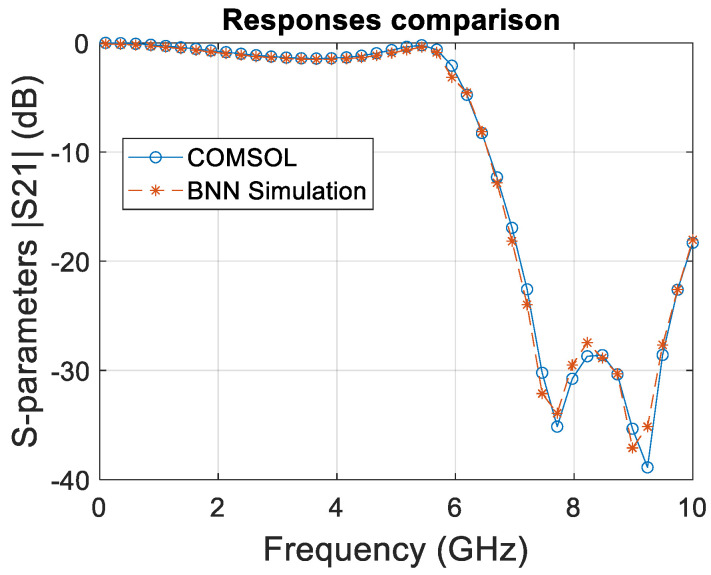
Corresponding comparative analysis of S21 transmission coefficients from COMSOL and BNN coarse model prediction from 0.1 to 10 GHz.

Both the COMSOL and BNN curves shown in Figure 8 closely follow each other, which indicates a high degree of correlation between the simulated data and the BNN predictions.

This suggests that the BNN effectively learned the underlying physical phenomena governing the system’s behavior over the frequency sweep. The BNN successfully captured the resonance peaks, which are critical in filter design as they represent the frequencies at which the filter significantly attenuates the signal. Accuracy in predicting these peaks is essential for the validation of the neural network model in practical applications.

### 5.2. Advanced Design Predictive Accuracy

**Figure 9:**
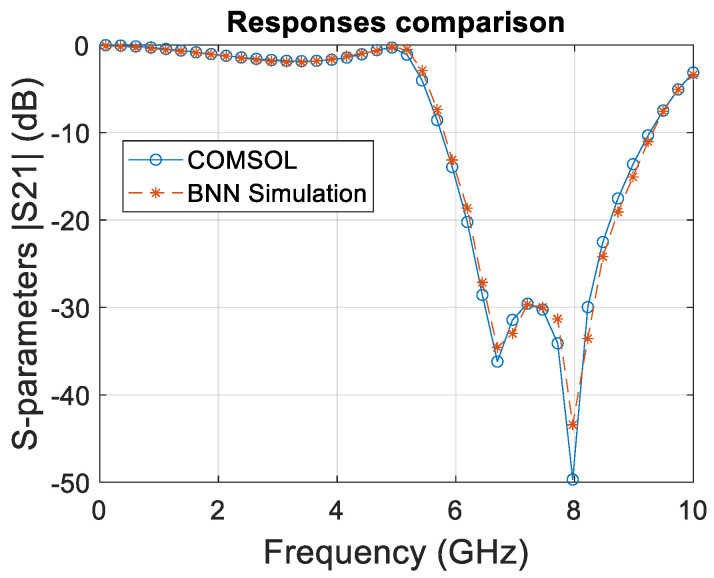
Corresponding comparative analysis of S21 transmission coefficients from COMSOL and BNN fine-model prediction from 0.1 to 10 GHz.

This new study extended the BNN framework to include temperature variations, enhancing the predictive accuracy for S21 transmission coefficients within the RF spectrum. The comparative analysis presented in Figure 9, spanning a frequency range of 0.1–10 GHz, demonstrates a remarkable alignment between BNN outputs and COMSOL simulations, reinforcing the BNN’s ability to internalize and replicate complex EM behaviors.

The graph meticulously compares the S21 transmission coefficients, a crucial performance metric obtained from the esteemed COMSOL multiphysics 6.0 software, against those predicted by the BNN model. The congruence of the curves throughout the frequency sweep, especially at critical resonance frequencies, signifies not only the precision of the BNN but also its potential as a tool for rapid prototyping and iterative design processes. This study underscores the significance of incorporating multiphysical parameters, such as temperature, in the training dataset to mirror the accuracy of traditional simulation methods while surpassing their efficiency.

Such fidelity in predictive modeling heralds a transformative era in EM simulation, where BNNs can expedite the design cycle without the need to compromise on the accuracy of resonance peak predictions. This leap in simulation technology could redefine the efficiency of EM filter design, with direct implications for industries that rely on precise and rapid development cycles.

The slight deviations observed between the BNN predictions and COMSOL results suggest an avenue for further refinement, potentially through enhanced neural network architectures or deeper training sets encompassing broader operational conditions. The validation of the BNN training regimen sets a precedent for the future integration of machine learning within EM analysis, promising significant strides in predictive accuracy and simulation swiftness.

## 6. Discussion: Bridging Theoretical Models with Advanced Multiphysics Simulations

Our investigation has unfolded a methodical and evolutionary strategy in the realm of filter optimization, transitioning seamlessly from foundational electromagnetic analysis to sophisticated multiphysics modeling. By weaving thermal and mechanical dynamics into the fabric of our design process, we have not only augmented the predictive accuracy of our models but also set new standards for filter optimization amidst the complexities of operational environments. This advancement heralds a paradigm shift towards a more nuanced and adaptive optimization strategy, laying the groundwork for future explorations into the integration of comprehensive multiphysics simulations into electronic system design.

The empirical evidence from our study showcases a remarkable alignment between the BNN predictions and COMSOL multiphysics simulations, affirming the robustness and validity of our integrated BNN-LHS approach. This synergy not only highlights the precision of our methodology but also opens avenues for its application in refining the design and optimization processes of electromagnetic components. When juxtaposed with conventional methodologies, our approach emerges as superior, offering unmatched accuracy and efficiency.

A pivotal aspect of our findings is the model’s fidelity across the entire frequency spectrum, adeptly capturing the nuanced behaviors of the filter in both passband and stopband regions. The BNN model excels in delineating the filter’s response, especially in areas of rapid S21 response changes, such as the steep transitions into and out of resonance valleys. This capability underscores a well-calibrated model that intricately understands the frequency-dependent characteristics of the filter, showcasing the potential of machine learning to capture complex physical phenomena.

Despite the overall high congruence, any deviations, however minor, between the BNN predictions and COMSOL simulations warrant attention. These discrepancies serve as indicators for potential areas of model enhancement, suggesting the need for additional training or possibly the development of a more intricate neural network architecture to fully encapsulate the physical system’s complexity.

The congruence between BNN predictions and COMSOL responses validates our model training regimen, suggesting that the selected architecture, learning rate, and training duration were aptly chosen for this predictive task. The BNN’s proficiency in mirroring COMSOL outcomes can revolutionize the design process, enabling rapid, accurate evaluations of system performance across various frequencies without relying solely on time-consuming physical simulations.

## 7. Conclusions: Pioneering the Future of Electromagnetic System Analysis with Advanced Computational Models

This investigation represents a pivotal advancement in EM system analysis, illustrating the transformative power of BNNs when integrated with LHS and further enriched by thermal dynamics. Our methodological breakthrough, which skillfully merged BNNs with LHS, has not only refined the accuracy of EM characterizations but also ushered in a new era of computational efficiency, redefining traditional design and optimization processes. The strategic implementation of nuanced parameter adjustments and sophisticated sampling strategies, as well as the inclusion of thermal variables, was instrumental in surpassing the limitations of conventional EM analysis methods.

The initial phase of our research highlighted the robustness of the BNN-LHS framework in enhancing the design and simulation of RF low-pass filters, setting a new standard for methodological precision and computational speed. This foundational phase paved the way for a more comprehensive exploration that embraced a multiphysics approach, incorporating thermal and mechanical dynamics into EM simulations. This ambitious endeavor has markedly deepened our understanding of filter behavior, marking a significant leap in both simulation accuracy and operational efficiency.

Our results not only affirm the BNN model’s exceptional predictive accuracy across an extensive frequency spectrum, but also underscore its capability to adeptly generalize from a rich dataset to novel scenarios. This breakthrough heralds a shift towards swift, data-driven filter optimization, promising unprecedented levels of efficiency and precision in EM system design. The seamless alignment of our predictive outcomes with established COMSOL simulations validates our integrated approach, establishing a new benchmark for predictive modeling within the EM domain.

As we look to the future, the fusion of machine learning with computational physics, especially through the integration of thermal dynamics, unveils expansive opportunities for research and innovation. Our contributions extend beyond enhancing the current understanding of multiphysical interactions in filter performance; they lay the groundwork for the development of next-generation simulation methodologies. The potential for the further exploration and application of these advanced, data-driven approaches is limitless; these technologies are poised to redefine the design, optimization, and characterization of complex EM systems under variable environmental conditions.

In summation, this study makes a profound contribution to both the theoretical and practical realms of EM system characterization, emphasizing the need for ongoing refinement and the pursuit of innovative, data-driven techniques for model improvement. Standing at the threshold of technological advancement, our findings light the way for future innovations in electronic systems, envisioning a future where the synergy of machine learning and computational physics catalyzes the evolution of electronic system design and manufacturing.

## Figures and Tables

**Table 1:** Design parameters for COMSOL-simulated low-pass filter.

Simulation Parameter Description	Coarse Model	Fine Model
Smallest Mesh Element Size Overall	0.397 mm	0.397 mm
Largest Mesh Element Size Overall	7.94 mm	7.94 mm
Minimum Mesh Size in Microstrip Line	1.667 mm	0.2504 mm
Maximum Mesh Size in Microstrip Line	8.3352 mm	1.2518 mm
Smallest Mesh Size in Port Regions	0.2794 mm	0.2794 mm
Largest Mesh Size in Port Regions	2.79 mm	2.79 mm

**Table 2:** Design parameters for COMSOL-simulated low-pass filter.

Parameter Description	Value	Identifier
Dielectric constant	2.2	$\varepsilon_{r}$
Substrate layer thickness	0.794 mm	H
Input/output trace width	2.45 mm	$w_{p}$
Input/output trace length	10 mm	$L_{p}$
Gap width between microstrip lines	3.5 mm	$W_{1}$
Length of open circuit stubs	5.6 mm	$L_{1}$
Distance between feeding stubs	4.2 mm	$S_{1}$
Proximity to front edge of substrate	0.5635 mm	$x_{gap}$
Proximity to side edge of substrate	6.6681 mm	$y_{gap}$
Height above ground plane	9.528 mm	$H_{air}$

**Table 3:** Phase-I: BNN’s performance sorted from the best to worst testing generalization MSE across dimensional variations.

Variation	MSE	MLE (%)	MTE (%)	Neurons
0.15	0.2903	11.3117	15.9565	13
0.2	0.3365	30.4209	36.0903	11
0.1	0.7454	12.3728	35.9268	10
0.25	0.8921	23.4974	38.28	10
0.3	2.6578	24.1204	47.856	13

**Table 4:** Phase-II: BNN’s performance sorted from the best to worst testing generalization MSE across dimensional variations.

Variation	MSE	MLE (%)	MTE (%)	Neurons
0.1	0.4083	17.7516	31.9933	11
0.2	0.4699	20.3328	35.2395	13
0.25	0.4855	11.6722	33.3739	15
0.15	0.4993	13.0466	25.4227	14
0.3	1.428	24.6704	35.8113	12

## Data Availability

The raw data supporting the conclusions of this article will be made available by the authors on request.
